# The dehydrins gene expression differs across ecotypes in Norway spruce and relates to weather fluctuations

**DOI:** 10.1038/s41598-020-76900-x

**Published:** 2020-11-27

**Authors:** Jaroslav Čepl, Jan Stejskal, Jiří Korecký, Jakub Hejtmánek, Zuzana Faltinová, Milan Lstibůrek, Salvador Gezan

**Affiliations:** 1 Faculty of Forestry and Wood Sciences, Czech University of Life Sciences, Kamýcká 1176, 165 21 Praha 6, Suchdol, Czech Republic; 2 VSN International, Hamel Hamstead, UK

**Keywords:** Gene expression, Genetics, Forest ecology

## Abstract

Norway spruce has a broad natural distribution range, which results in a substantial variety of its physiological and genetic variation. There are three distinct altitudinal ecotypes described in this tree species. The physiological optimum of each ecotype may be shifted due to ongoing climate change, especially in traits associated with water demand that might be crucial for adaptation. Dehydrins are proteins that help to mitigate the adverse effects of dehydration. Dehydrin gene expression patterns appeared to be a suitable marker for plant stress assessment. Genetically determined differences in response between individuals and populations were formerly studied, however, mainly in controlled conditions. We evaluated ecotypic variation in dehydrin gene expression in a clonal bank comprised of all three ecotypes. A genetic relationship among targeted trees was uncovered utilizing GBS (Genotyping by Sequencing) platform. We sampled 4–6 trees of each ecotype throughout 15 months period. Subsequently, we assessed the RNA expression of dehydrin genes by qRT-PCR. For this study, we deliberately selected dehydrins from different categories. Our findings detected significant differences among ecotypes in dehydrin expression. The association of recorded climatic variables and individual gene expression across the study period was evaluated and revealed, for certain genes, a correlation between dehydrin gene expression and precipitation, temperature, and day-length.

## Introduction

Norway spruce (*Picea abies* [L.] Karst.) is a dominant species in mountainous, sub-alpine, and boreal forest ecosystems. Its large natural range extends from the French Alps in the west (5°E longitude) to the Ural Mountains (155°E) in the east and from 70°N latitude in northern Norway to 42°N in Macedonia^1^. Its altitudinal range stretches from sea level to above 2300 m in the Italian Alps^2^. Norway spruce is one of the essential coniferous tree species of the European continent in terms of wood and pulp production.

Owing to Norway spruce broad biogeographic range, natural selection has resulted in a substantial genetic diversity. However, high intra-population variability was conveyed to the extent surpassing the inter-population variability^3^.

In general, three morphological forms of Norway spruce are distinguished. Those forms also differ in their ecological requirements and are tightly connected to their altitudinal origin^4^. Wide crown, toothed cone scales, and comb-like branches characterize the low–elevation form *acuminata* (naturally growing up to 500 m a.s.l. in Central Europe). The high-elevation form (*obovata*; naturally growing approx. above 1100 m a.s.l.) has a typically narrow crown, round cone scales, and flat branches. It usually occurs close to the tree line. The medium-elevation form (*europaea*) has a rhombic, toothed cone scales with crown mostly conical of medium width. Its branches exhibit a brush type, and they are usually shorter, dense, and hanging^4^. Moreover, for each ecotypic form, specific adaptations for the given geographic area, climatic area, and forest vegetation zone are reported^4–6^.

With ongoing climate change, various models predict rapid shifts in microclimatic conditions along longitudinal and altitudinal gradients. Current local adaptations may not be sufficient for future forests^7^. Namely, traits connected to water demand are crucial for adaptation as they are strongly affected by climate change, and Norway spruce is sensitive to drought stress^8^. Drought-induced stress is considered a cardinal factor connected with an ongoing bark beetle outbreak and possibly leads to Norway spruce decline across the Central European region. On the contrary, high adaptive variation in drought-related traits within Norway spruce populations has been described^9^.

Lately, stress adaptive traits have been investigated through the gene expression analysis of dehydrins, proteins of the late embryogenesis abundant (LEA) family. Conditions of drought, low temperatures, frost, or high salinity have been associated with dehydrin gene expression^10–13^. It has been described that dehydrins maintain or mimic aqueous conditions in a dehydrated cell^14,15^. Dehydrin proteins are divided into categories differing in the presence and order of typical amino acid motifs. Conserved K-segment is present in all dehydrins; other commonly described motifs are S-segment and Y-segment^16^. Using the abbreviation YSK according to the quantity and sequence of segments Y, S, K, the dehydrins can be sorted into subcategories^11^.

Dehydrin gene expression analyses are frequently reported in economically important horticultural species^17–19^. However, information on genetic variation in dehydrin gene expression is scarce in gymnosperm species. A study of dehydrin gene expression in *Pinus pinaster* revealed that expression patterns of several dehydrin transcripts differed significantly among various provenances. And even more contrasting results were observed between drought-sensitive and drought-tolerant genotypes^20^. The dehydrin gene expression of three *Picea glauca* genotypes detected only minor differences in single dehydrin gene expression (PgDhn10)^21^*.* Such experiments have been carried out in controlled conditions (tissue cultures, greenhouse planting, or growth chambers). In contrast, knowledge about genetically determined differences in dehydrin expression in forest trees grown in natural conditions is scarce.

Our study aimed to evaluate variation among Norway spruce ecotypic forms in dehydrin gene expression. We conducted our research in a Norway spruce clone bank, consisting of all three ecotypes. For this study, we selected three dehydrins from different categories (Kn, SKn, and KnSKn): PaDhn4.5 from SK_2_ subcategory, PaDhn6 from K_3_ subcategory, and PaCAP1.1 from subcategory K_4_SK_2_. To justify clustering into ecotypic groups objectively, we further reconstructed a genetic relationship among sampled trees utilizing Genotyping by Sequencing (GBS). Besides, the association of recorded climatic variables with the specific gene expression across the study period was evaluated.

## Material and methods

### Plant material and forest stand description

The original field trial corresponded to a Norway spruce clone bank, established in 1970 with clonally propagated accessions originating from several autochthonous Czech Norway spruce populations^22^. The plot was planted as clonal rows with ten individuals per clone in 3 m distances; distances between rows were 6 m. The whole site was subsequently pruned so that the final spacing is 6 m × 6 m. The trial is located in the central part of the Czech Republic (N 49°56.37′, E 14°20.96′), situated on a mild NW slope with a low relief of an altitude of 320–345 m a.s.l. The average precipitation (mean between years 1980–2016) in the area was 587 mm, with an average annual temperature of 8.6 °C.

Bedrock is constituted from clayey Algonkian phyllite slates with variously thick loess and sloping clay overlaps. The soils can be characterized as medium-deep cambisols in strongly skeletal bases, in places with signs of reduction processes. The upper horizons are clayey, the lower horizons heavier, silty clay. There is a lack of loess cover in places, and the soils are generally rich in the skeleton (particles > 2 mm). The average tree height on the plot was 20.6 m; sd = 2.95 m, and the average DBH was 33.1 cm; sd = 7.7 cm.

The sampling procedure consisted of randomly selecting strips from a given ecotype. Within these strips, trees were chosen randomly, ensuring that we cover different microsites within the trial. All sampled trees retained their morphological characteristics (ecotypic appearance) correspondingly to their altitudinal origin. The typical appearance of ecotypic forms is displayed in Supplementary Figure S1.

Trees (*N* = 4) representing a low-elevation form (*acuminata)* came from an altitude of 360 m a.s.l., trees (*N* = 6) belonging to medium-elevation form (*europaea)* originated from elevation of 770–775 m a.s.l., whereas trees (*N* = 4) representing high-elevation form (*obovata)* originated from stand at altitude of 1145–1175 m a.s.l.

Samples were collected monthly through April to December 2016, and in February, March, April, and June 2017. We targeted last-year needles from the middle part of the crown (8–10 m above ground) from the southern direction. Branches were cut with pole-scissors. Samples were immediately placed into liquid nitrogen in 50 mL Falcon vials, then stored in the freezer (− 80 °C) until further processing.

### Climatic data

Reported climatic variables (daily temperature and its extremes, day length, sunshine duration, daily mean precipitation, and air humidity) were obtained from the nearest state meteorological station (Praha–Libuš), located approx. 10 km away from the study site.

### Laboratory analysis

For the RNA isolation, needles (100 mg) were put into 2 mL Eppendorf Safe-Lock tubes containing three steel grinding balls and frozen under liquid nitrogen. Subsequently, the tissue was ground with Retsch Mixer Mill 400. Total RNA was extracted with Epicentre MasterPure RNA Purification Kit (Epicentre). RNA was quantified by a 260/280 nm absorbance ratio using the NanoDrop spectrophotometer (Thermo-Fisher Scientific). Complementary DNA (cDNA) was synthesized with the High Capacity cDNA Reverse Transcription Kit (Thermo-Fisher Scientific) according to the manufacturer's instructions. The final volume of 40 µl cDNA solution was further diluted to 160 µl volume.

Samples for DNA extraction were treated similarly—100 mg plant tissue was ground under nitrogen liquid. Genomic DNA was extracted using the DNeasy Plant Mini Kit (Qiagen) following the manufacturer's instructions, and the DNA product was evaluated by spectrophotometrical measurement. After initial quality control, DNA samples were shipped to Cornell University Biotechnology Resource Center (BRC) and genotyped on Genotyping by Sequencing platform^23^.

### Quantitative real-time PCR (qRT-PCR)

Specific primers for reference actin gene and dehydrin genes PaDhn6, PaDhn4.5, and PaCAP1 were designed according to^24^. Oligonucleotides were synthesized by Eurofins MWG Operon (Ebersberg, Germany). The specific dehydrin sequences were previously identified as plausible sensitive markers of drought stress in Norway spruce^13^.

Quantitative real-time polymerase chain reaction (qRT-PCR) was performed in a 12-μl volume containing 250 nM of reverse and forward primer, 2 µg of cDNA, 500 nM ROX (Top-Bio), and 6 μl of SYBR Green PCR Master Mix (Top-Bio) using the StepOnePlus Real-Time PCR System (Applied Biosystems) with the parameters recommended by the manufacturer. Reaction conditions were set as 2 min at 50 °C, 10 min at 95 °C, and 25 cycles of 95 °C for 15 s, 72 °C for 20 s and 63 °C for 30 s. Each PCR reaction was run in triplicates on a plate, and each plate was duplicated. The threshold cycle (Ct) values were calculated by StepOnePlus software (Applied Biosystems). All the gene expression levels were normalized to actin gene expression, chosen as endogenous control^24,25^. Dhn4.5 to Dhn6 ratio was calculated according to^13^.

### Statistical analysis

For statistical analysis, the R statistical software^26^ was utilized. Pearson's product-moment correlation coefficients and their statistical significance were assessed using the 'cor.test' function, as implemented in R. The mean of 14 days that preceded sampling was calculated for all analyzed climatic variables and subsequently correlated to relative dehydrin gene expression. For visualization, Locally Estimated Scatterplot Smoothing (LOESS) was utilized with a span set on 0.75.

We fitted a univariate linear mixed model was fitted to evaluate dehydrin gene expression considering repeated measurements with the terms:

1 $$Y = {\mathbf{1}}{\varvec{\mu}} + {\varvec{X}}_{{\mathbf{1}}} {\varvec{a}} + {\varvec{X}}_{{\mathbf{2}}} {\varvec{b}} + {\varvec{X}}_{{\mathbf{3}}} {\varvec{ba}} + {\varvec{e}}$$

where ***Y*** corresponds to the data vector; *μ* is the overall mean effect; ***a*** is the fixed vector of individual ecotypes; ***b*** is the fixed vector of repeated events (months), ***ba*** is a fixed vector of the interaction of ecotypes with months, and ***e*** is the random vector of errors, with

2 $${\varvec{e}} \sim MVN\left( {{\mathbf{0}},{\varvec{I}}_{{\varvec{n}}} \otimes {\varvec{R}}} \right),$$

***R*** is a 13 × 13 matrix of variance–covariance components for residuals defined as a uniform correlation among measurements for the same individual but different variances per month, and ***In*** is an identity matrix identifying each tree. The letters ***X*** designate incidence matrices for all fixed effects, and ***1*** is a vector of ones.

### Genomic data acquisition and analysis

As a prerequisite for dehydrin gene expression analysis, we evaluated genomic profiles of targeted trees. Genotyping by sequencing (GBS) is an NGS-based platform highly suitable for large genome species as the genome complexity is reduced, and the genome coverage enhanced, using restriction enzymes treatment.

Genomic data in VCF file format (approx. 9 Mio SNPs of raw data) obtained from BRC was filtered in VCF Tools software^27^ to keep only biallelic loci with the minor allele frequency higher than 0.01 and mean depth values between 20 to 80. From here, ~ 6 k high-quality SNPs were retained for subsequent analysis. Discriminant analysis of principal components (DAPC) implemented in R package adegenet 2.1.0^28^ was used for genetic clustering. These methods allowed us to assess the putative ecotypes based not only on historical records but also on real genetic similarity among individuals.

## Results

### Genetic analysis of Norway spruce ecotypic forms

**Figure 1:**
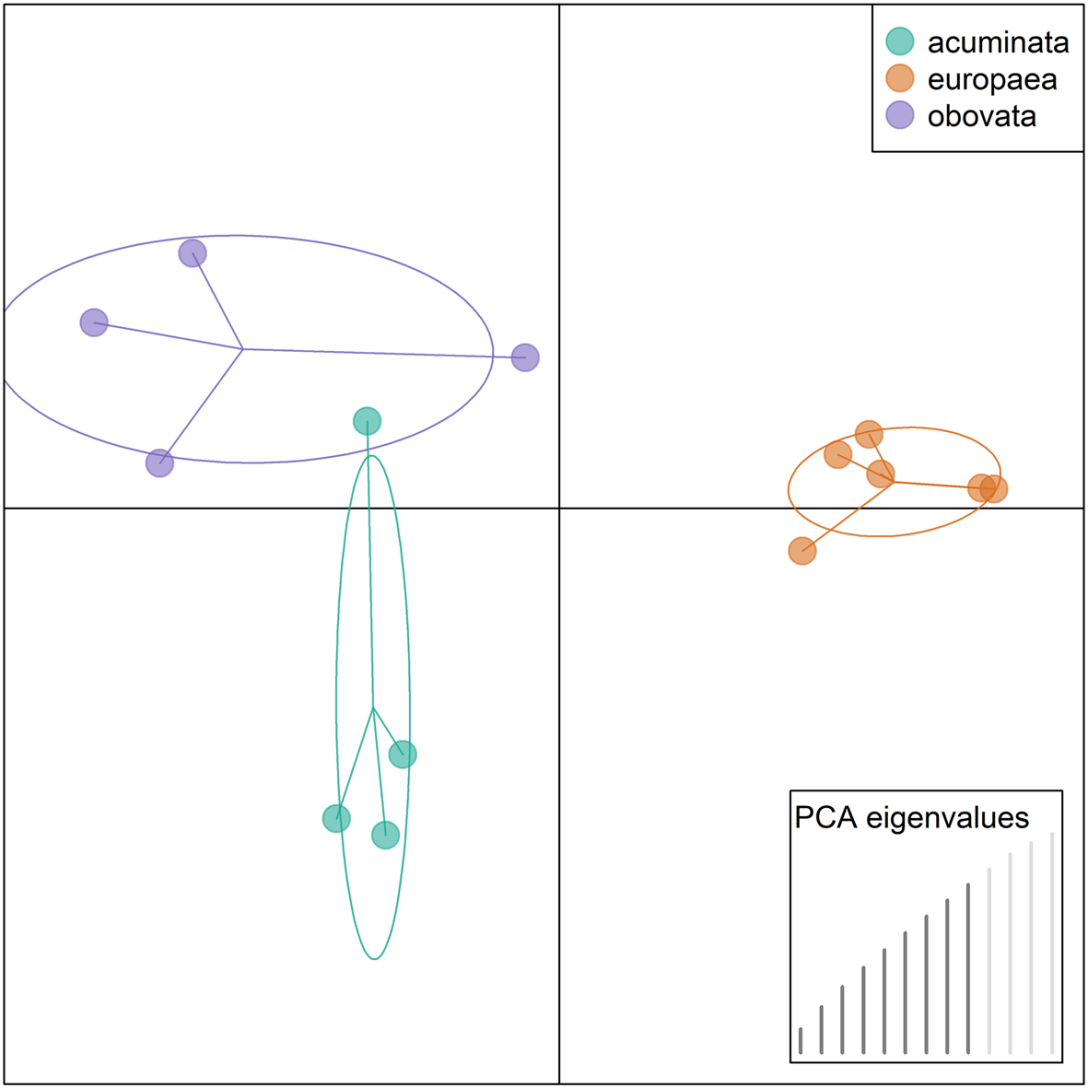
DAPC analysis differentiated individuals into respective forms.

As grouping into three ecotypic units is based on phenotypic appearance (mostly on crown morphology), we evaluated these groups with a comparative genetic analysis (Fig. 1). Our results indicate that even though the sample size is insufficient for accurate cluster analysis, we can verify genetic proximity among individuals belonging to the same ecotypic groups and the distinct genetic structure among these forms, respectively. The genetic distance of medium-elevated ecotype (*europaea*) is, interestingly, the most delimited compared to the other two groups (Fig. 1).

### Differences in dehydrin genes expression between ecotypic groups

**Figure 2:**
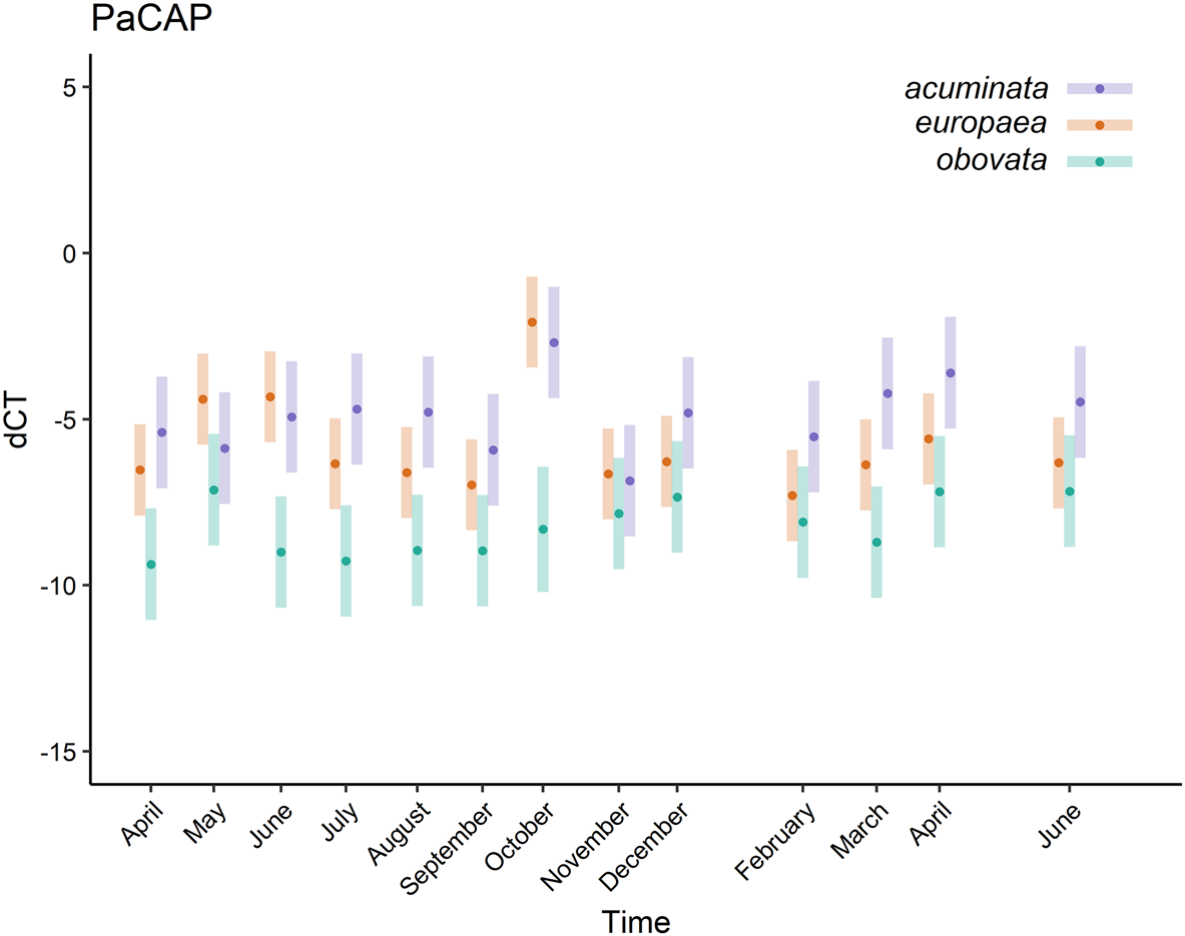
Mean relative dehydrin PaCAP expression of different ecotypes throughout the 15-months; 95% confidence interval indicated.

A repeated measurement assessed differences in dehydrin gene expression among Norway spruce ecotypes in a span of 15 months. Statistically non-significant differences in the expression of PaDHN4.5 and PaDHN6 genes among observed ecotypes were found, whereas statistically significant differences among ecotypes were detected in PaCAP1 gene expression (Fig. 2).

Furthermore, differences in individual months in PaCAP1 dehydrin gene expression were analyzed by Duncan's multiple range test. No differences between *acuminata* and *europaea* ecotypic forms were detected during the monitored time-span. In contrast, significant differences were found between both *acuminata/obovata* and *europaea/obovata* ecotypic forms. Diverging expression appeared most frequently between *acuminata* and *obovata* originating from the most distant locations in terms of altitude (Table 1).

**Table 1:** Differences in PaCAP expression (*p* values) between ecotypes in individual months.

	Apr. 2016	May 2016	Jun. 2016	Jul. 2016	Aug. 2016	Sept. 2016	Oct. 2016	Nov. 2016	Dec. 2016	Feb. 2017	Mar. 2017	Apr. 2017	Jun. 2017
*A–E*	0.308	0.184	0.587	0.139	0.101	0.343	0.577	0.851	0.187	0.112	0.054	0.074	0.100
*A–O*	**0.001**	0.302	**0.001**	** < 0.001**	** < 0.001**	**0.013**	** < 0.001**	0.416	**0.038**	**0.035**	** < 0.001**	**0.004**	**0.028**
*E–O*	**0.011**	**0.015**	** < 0.001**	**0.009**	**0.036**	0.075	** < 0.001**	0.282	0.336	0.469	**0.037**	0.153	0.441

Ecotypes are denoted as *A—acuminata; E—europaea* and *O—obovata.* Significant differences (alpha > 0.05 level) are in bold.

### Recorded climatic variables versus dehydrin expression

**Figure 3:**
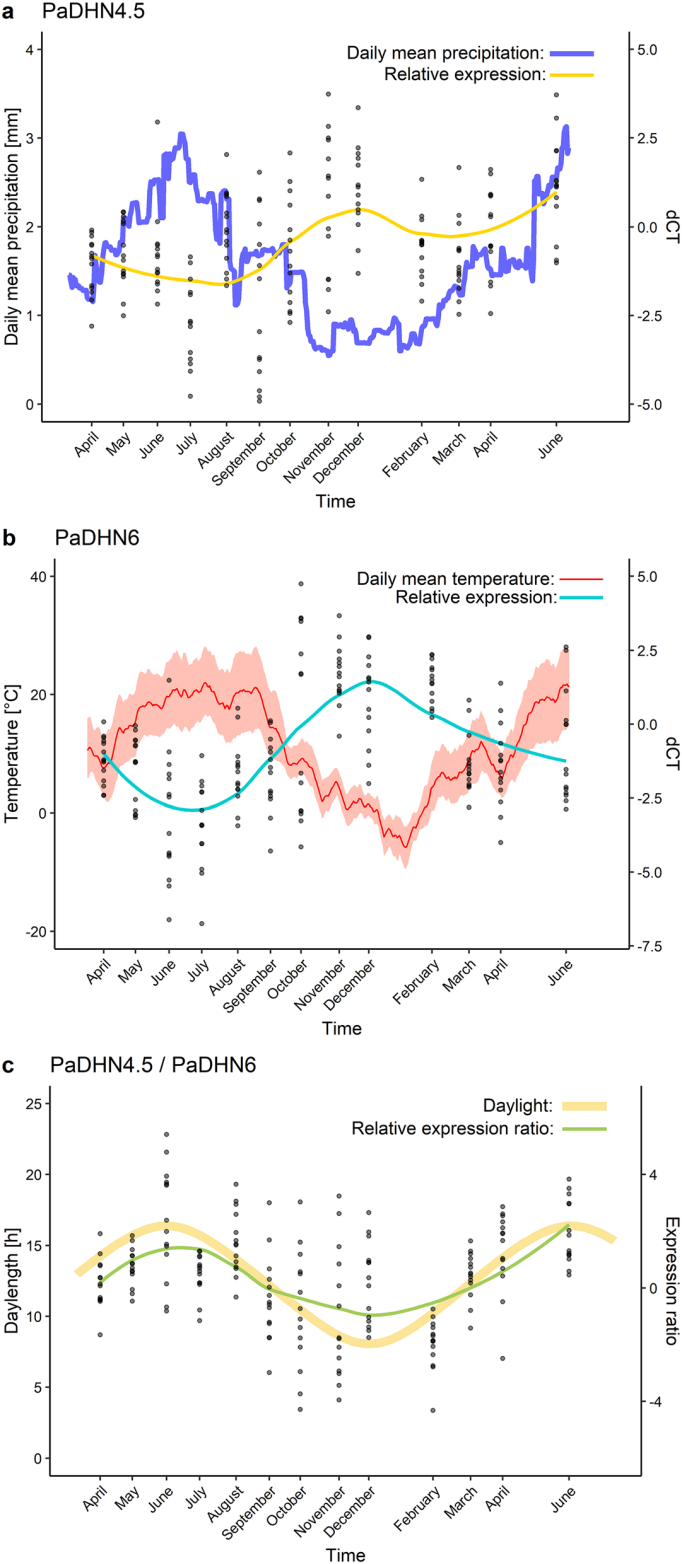
Relative dehydrin expression is connected to climatic variables. (**a**) yellow line depicts the LOESS curve of PaDHN4.5 expression related to mean daily precipitation (mm) averaged across 30 day period; (**b**) blue line represents the LOESS curve of PaDHN6 expression related to observed trend in temperatures throughout the 15 months, the red line follows mean daily temperature (°C) averaged across 14 days periods, the interval of corresponding max and min daily temperatures is illustrated in pink ribbon; (**c**) green line depicts LOESS curve of PaDHN4.5/PaDHN6 expression ratio, the yellow line represents day-length (h); black dots display the relative expression of 14 samples.

Mean values of PaDhn4.5 and PaDhn6 relative gene expression in given ecotypic forms were not significantly different throughout the measured period. With this in mind, the expression data of various ecotypes were pooled for posterior correlation study. PaCAP1 relative expression displayed significant differences among ecotypic forms, as reported above. For this reason, each ecotype was considered separately within the correlation study. The relation of dehydrin expression to climatic variables is outlined in Table 2, and detailed patterns of notable correlations are depicted in Fig. 3.

**Table 2:** Correlations of climatic variables with dehydrin expressions.

Climatic variable	PaDhn4.5		PaDhn6		PaCAP						PaDhn4.5/PaDhn6	
	cor	*p*	cor	*p*	*acuminata*		*europaea*		*obovata*		cor	*p*
					cor	*p*	cor	*p*	cor	*p*		
Mean daily temperature	− 0.40	0.179	− **0.81**	**0.001**	0.03	0.911	0.07	0.818	− 0.35	0.242	**0.66**	**0.015**
Max. daily temperature	− 0.41	0.164	− **0.82**	**0.001**	0.02	0.946	0.03	0.910	− 0.38	0.205	**0.66**	**0.015**
Min. daily temperature	− 0.44	0.136	− **0.82**	**0.001**	0.07	0.818	0.16	0.606	− 0.35	0.241	**0.64**	**0.018**
Day length	− 0.35	0.245	− **0.86**	** < 0.001**	0.13	0.671	0.14	0.639	− 0.29	0.328	**0.76**	**0.002**
Sunshine	− 0.13	0.673	− 0.54	0.056	− 0.11	0.726	− 0.20	0.523	− 0.16	0.596	**0.57**	**0.043**
Air humidity	0.17	0.576	**0.69**	**0.009**	− 0.10	0.740	0.08	0.795	0.12	0.701	− **0.71**	**0.006**
Mean daily precipitation	− **0.64**	**0.018**	− **0.63**	**0.022**	− 0.11	0.732	− 0.04	0.905	− **0.62**	**0.025**	0.20	0.511

‘cor’ stands for Pearson's correlation coefficient and ‘*p*’ for a respective *p*-value. Significant correlations (alpha > 0.05 level) are in bold.

A significant negative correlation (r = −0.64) between PaDhn4.5 expression and mean daily precipitation was found in our dataset, whereas all the other remaining climatic variables did not significantly correlate with PaDhn4.5 expression.

A relatively strong negative correlation between daily temperature (mean, max, min) and PaDhn6 expression level (− 0.82 on average) was observed. However, the strongest negative correlation (− 0.86) was found with day length. Relative expression of PaDhn6 negatively correlated also with precipitation (− 0.63). On the other hand, a moderately positive correlation was observed with air humidity (0.69).

The significant negative correlation between PaCAP1 expression and precipitation was found only for *obovata* ecotype (− 0.61).

## Discussion

We assume that Norway spruce ecotypes in the Czech Republic represent several populations sharing a similar set of adaptations. In our case, trees belonging to their respective ecotypes appear to be more alike based on SNPs data analysis. Although sampled trees have been growing under similar conditions, they retained their ecotypic membership's significant morphological features. Thus, we expected that each ecotype's divergent adaptations would lead to the different dehydrin gene expression, as the same environmental signals will be interpreted differently.

Consistently with our primitive assumption, we detected significant differences among ecotypic forms in PaCAP1 expression. The *acuminata* (low-elevation) form displayed the highest relative expression across the sampling period, whereas the *obovata* (high-elevation) form showed the lowest one. Finally, the *europaea* (medium-elevation) form showed the intermediate values; however, it was closer to *acuminata*. These differences might be attributable to a more extreme high elevation environment of *obovata* and its specific adaptation. These adaptations might have been crucial for pushing up the threshold of stress levels necessary for increasing the dehydrin expression.

It should be noted that PaCAP1 dehydrin expression did not appear to be driven by climatic variables—only a moderate negative correlation between precipitation and PaCAP1 in *obovata* ecotypic form was found. In accordance, a significant difference between early and late flushing families of Norway Spruce in PaCAP1 expression was observed^12^.

Contrastingly, these ecotypic differences were not found in PaDhn4.5 and PaDhn6 gene expression. It may be explained by the acclimation of trees to given stand conditions resulting in homogeneous stress response as described by^29^. An alternative explanation could be that gene expression of these dehydrins exhibits a low variation within the species, and a higher number of individuals would have to be analyzed to detect minor differences.

### Dehydrin expression concerning annual climatic shifts

To protect themselves from frost's adverse effects in the winter, temperate perennial plants typically enter dormancy and cold hardiness. This period is connected with photosynthesis cession, alteration of carbon metabolism, and cryo- and osmoprotectants synthesis. LEA proteins and proline are considered the stress proteins with osmoprotective function. Dehydrins, as a part of the LEA proteins family, can attract common stress-signaling phospholipids^30^. The commonly mentioned protecting the binding activity of dehydrins to the proteins was not proved. However, a very similar process could be expected because many polar amino acids are part of the dehydrins.

Nevertheless, the plant usually involves many other substances that lower the plant water potential and maintain the cell's water molecules, known as osmoprotectants. These organic, highly soluble, and electrically neutral substances could be non-reducing sugars and sugar alcohols (e.g., trehalose or inositol), betaines (e.g., glycine betaine), and amino acids as proline^31^. Many sugar osmoprotectants play several essential roles in the plant as the growth regulator and signal molecule and can be participants in the communication network within a plant^32,33^.

We documented the relation of dehydrin genes expression with climatic variables across the 15 months sampling period. As reported in other studies, levels of dehydrin gene expression in forest trees were associated with winter dormancy^34–36^, bud burst^12^, and with both temperature and photoperiod^37–39^. Furthermore, it was documented that dehydrin gene expression is under the influence of temperature in Norway spruce^25,40^. We are bringing a new insight into the problem in the context of the unique common-garden experiment in a prolonged time-span. This setting allowed various environmental impacts on the studied material. In the next section, we will discuss individual dehydrin genes expression patterns related to climatic conditions.

### PaDhn4.5 from SK_2_ subcategory

The PaDhn4.5 relative expression was reported to be regulated by water status and dormancy level^12^ and decreased correspondingly in the spring months from April to June. PaDhn4.5 expression was significantly downregulated in trees undergoing drought stress^13^. Following these findings, we observed the lowest value of the relative expression for all three ecotypes in July 2016 during the longer-lasting dry period. The opposite trend occurred within months with the highest precipitation (August, November, and December 2016), when relative expression values oscillated around their maxima (see Fig. 3b). Exception from this trend can be noted in June 2017, where both precipitation and PaDhn4.5 expression were high. However, across the evaluation period, a significant negative correlation (r = −0.64) of PaDhn4.5 expression with precipitation was present, whereas all the other remaining climatic variables did not significantly correlate with PaDhn4.5 expression. These findings may suggest that the relative expression of PaDhn4.5 responds primarily to drought stress.

### PaDhn6 from K_3_ subcategory

Similarly, PaDhn6 was described as a drought stress-responsive marker^13^ with higher expression during drought periods. It was also shown that both day length and minimum temperature are regulation factors^41,42^. The decreasing PaDhn6 transcript level was observed from April to June^12^. We noticed the same trends in our results, with a strong negative correlation (r = −0.82) of PaDhn6 expression levels with both daily mean temperature and recorded extremes.

Short days were hypothesized to trigger the same dehydrin expression response as cold or drought stress in forest trees^43^. In accordance, we recorded a strong negative correlation of dehydrin PaDhn6 expression with day length (r = −0.79). PaDhn6 was described to be highly up-regulated after even a short period of short days^12^.

### PaCAP1 from K_4_SK_2_ subcategory

PaCAP1 was described to be responsive only to severe drought stress^13^. It is related to drought resistance under high water loss. Up-regulated expression of homologous PoCAP1 was also recorded in extremely low temperatures in closely related Siberian spruce^25^. In our study, the recorded levels of stress probably did not reach such extreme levels, and therefore, PaCAP1 did not exhibit any significant variation across the evaluated period. Only a moderate negative correlation between precipitation and PaCAP1 in *obovata* ecotypic form was found in our study. In this regard, the estimated temporal correlation (r = 0.25 ± SE 0.10) among repeated measures across the study period is relatively self-explanatory because PaCAP1 expression was not responsive to temporal changes. The stable transcript level of the PaCAP1 gene during the period from April to June was also reported^12^.

### PaDhn6/PaDhn4.5—Expression ratio

In addition to various single dehydrin gene expression profiles, PaDhn6 to PaDhn4.5 expression ratio was suggested as a sensitive marker of plant drought stress in Norway spruce^13^. This relative expression ratio follows temperature with a significant positive correlation (Table 2).

A previously published study recorded the lowest values of the respective ratio during the relatively moist periods^13^. In our case, the highest precipitation was recorded in November 2016. This month was also connected with the low value in the respective ratio. We found a strong negative correlation with air humidity, but we did not detect a significant association with precipitation across the whole period (Table 2).

In contrast to all single dehydrin genes, the PaDhn6/PaDhn4.5 significantly positively correlated with day length and sunshine duration in our study, which could explain the strong negative correlation with air humidity.

## Conclusions

In this study, we analyzed dehydrin gene expression concerning Norway spruce variation based on ecotypic elevation forms planted *ex-situ* on a clone bank plot. We believe this unique setting makes our results interesting for a broader scientific community. Methodically similar research has been primarily performed under controlled conditions in greenhouses or growth chambers^13,21^, while such studies under natural conditions are scarce^12^. It is worth noting that all three ecotypic forms growing at the same site retained the specific morphological characteristics according to their origin based on particular altitudes. We confirmed the underlying genetic background of these morphologic features observing inherent grouping patterns based on SNPs data.

Dehydrin gene expression profiles were analyzed by a linear mixed model to evaluate dehydrin gene expression over the 15 months. Significant variation among Norway spruce ecotypic forms in dehydrin gene expression was found. The ecotypic forms differ in PaCAP1, where *acuminata* and *obovata* are the most different forms in terms of dehydrin gene expression. The other observed dehydrin genes do not exhibit significant variation in ecotypic forms. These differences might be connected to relatively stronger adaptations that the *obovata* ecotype acquired in its more extreme habitat.

PaDhn4.5 and PaDhn6 dehydrin genes correlate rather strongly with a range of climatic variables such as temperature and precipitation. In contrast, PaCAP1 expression did not appear to be driven by climatic variables: only a moderate negative correlation between precipitation and PaCAP1 in *obovata* ecotypic form was found.

## Supplementary information

Supplementary Figure 1.
