# Supplementary material for: The dehydrins gene expression differs across ecotypes in Norway spruce and relates to weather fluctuations
Source: Sci Rep. 2020 Nov 27;10:20789. doi: 10.1038/s41598-020-76900-x (PMC7695824; doi:10.1038/s41598-020-76900-x)
Supplement: Supplementary file 1 — Supplementary Figure 1. [file 41598_2020_76900_MOESM1_ESM.docx]

The dehydrins gene expression differs across ecotypes in Norway spruce and relates to weather fluctuations

Jaroslav Čepl, Jan Stejskal, Jiří Korecký, Jakub Hejtmánek, Zuzana Bínová, Milan Lstibůrek, Salvador Gezan


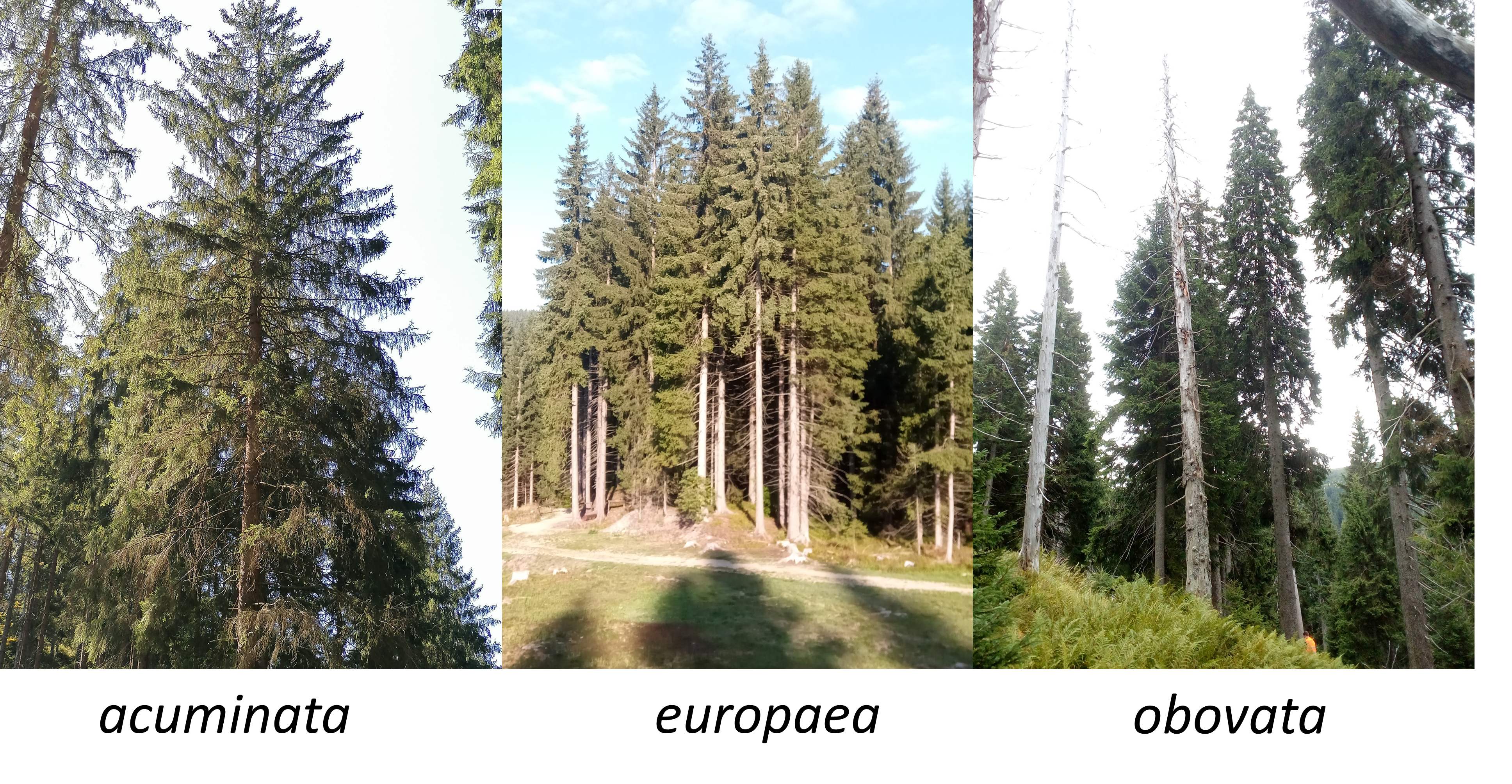


Supplementary Figure 1. Typical appearance of Norway spruce ecotypes (*author:* Jiří Korecký)
